# Sleep and Alcohol Use in Women

**DOI:** 10.35946/arcr.v40.2.13

**Published:** 2020-07-16

**Authors:** Sarah M. Inkelis, Brant P. Hasler, Fiona C. Baker

**Affiliations:** 1 Joint Doctoral Program in Clinical Psychology, San Diego State University and University of California San Diego, San Diego, California; 2 Department of Psychiatry, School of Medicine, University of Pittsburgh, Pittsburgh, Pennsylvania; 3 Center for Health Sciences, SRI International, Menlo Park, California; School of Physiology, University of the Witwatersrand, Johannesburg, South Africa

**Keywords:** adolescence, alcohol use disorder, circadian, sex differences, slow wave sleep, substance use

## Abstract

Sleep disturbance is common among individuals with alcohol use disorder (AUD). Insomnia not only is a pathway toward alcohol consumption but also is related to increased risk of relapse, psychosocial impairment, decreased quality of life, and suicidal ideation in individuals with AUD. Few studies examining sleep disturbance and alcohol use have explored how this relationship differs between men and women. Historically, studies of AUD have included few, if any, women in their samples. However, women are increasingly consuming alcohol at an earlier age and at higher rates, and the effect of alcohol on women’s mental and physical health is expected to rise. This narrative review consolidates findings from studies that have reported the effects of acute and chronic alcohol use on sleep among women. Additional research is needed to investigate sex differences in this area. Such research should consider the modifying effects of age, lifetime alcohol use, and psychiatric co-occurrence, as well as the effectiveness of combined interventions for AUD and sleep disturbance.

## INTRODUCTION

Sleep disturbance is one of the most common complaints of individuals with alcohol use disorder (AUD), with prevalence estimates ranging from 36% to 91%.^1^ Insomnia in particular has been associated with multiple aspects of AUD: relapse to drinking, psychosocial impairment (e.g., employment problems, social conflict, and impulse control), decreased quality of life, suicidal ideation, and insufficient sleep duration. (For definitions of insomnia and other technical terms, see the box **Glossary of Sleep Terms.**) Sleep disturbance can serve as a pathway to increased alcohol use, in part because alcohol can be used as a sleep aid to reduce time to sleep onset. However, even acute alcohol consumption increases sleep disruption throughout the night, and tolerance to the sedating qualities of alcohol accumulates quickly.^2^ In people with AUD, chronic alcohol use is related to changes in sleep structure that persist into abstinence. For abstinent individuals with AUD, this persistent sleep disturbance is a risk factor for relapse.^1^ Once relapse occurs, the cycle repeats, as continued consumption of alcohol perpetuates sleep disturbance.

**Glossary of Sleep Terms**

**Table:** 

**Actigraphy:**	An objective measure of sleep quantity and circadian patterns that uses an accelerometer (generally worn like a wristwatch) to detect sleep–wake activity over several days or weeks.
**Apnea-hypopnea index:**	An index used to indicate the severity of sleep apnea that is represented by the number of apnea and hypopnea events per hour of sleep.
**Circadian period:**	The amount of time for a cyclical process to return to the same phase (e.g., from one day’s waking to the next day’s waking).
**Circadian preference/chronotype:**	An individual’s tendency towards relatively earlier or relatively later sleep and activity patterns, typically measured via preferred timing (i.e., morningness versus eveningness) or self-reported actual timing (i.e., early versus late chronotype).
**Circadian rhythm:**	An endogenous 24-hour rhythm, typically measured via levels of melatonin or by core body temperature.
**Circadian timing:**	The timing of biological processes that follow a circadian rhythm (e.g., sleepiness, wakefulness, melatonin, body temperature).
**Hypopnea:**	The partial blockage of air, resulting in decreased airflow and oxygen saturation.
**Insomnia:**	A sleep disorder characterized by difficulty falling asleep or staying asleep, causing distress or impairment in daytime functioning.
**K-complex:**	A high-voltage delta frequency EEG event seen in NREM sleep that occurs when large numbers of healthy neurons fire in a synchronized manner.
**Non–rapid eye movement (NREM) sleep:**	The sleep stage characterized by slower, higher amplitude EEG activity, regular breathing and heart rate, muscle tone (i.e., low-level contraction), and a lack of eye movement; consists of stages N1, N2, and N3.
**Polysomnography (PSG):**	A test conducted to study sleep and diagnose sleep disorders using a multitude of physiological measures, including measures of brain activity, blood oxygen levels, heart rate, breathing, and muscle movements.
**Rapid eye movement (REM) sleep:**	The sleep stage characterized by low-amplitude, high-frequency EEG activity, rapid eye movement, irregular respiration and heart rate, and muscle atonia.
**Sleep apnea:**	A sleep disorder in which breathing is repeatedly interrupted during sleep.
**Sleep architecture:**	The structural organization of sleep, such as cyclical alternation of NREM and REM sleep stages.
**Sleep behavior:**	Self-report measures from questionnaires that typically ask about sleep over a period of weeks or months.
**Sleep-disordered breathing:**	An umbrella term that encompasses breathing disorders and respiratory abnormalities that occur during sleep, including sleep apnea and snoring.
**Sleep efficiency:**	The total number of minutes of sleep divided by the number of minutes in bed.
**Sleep electroencephalogram (EEG):**	A recording of brain activity during sleep.
**Sleep onset latency:**	The number of minutes to fall asleep after the lights are turned off.
**Sleep timing:**	The times of day an individual goes to sleep and wakes up.
**Slow wave activity:**	EEG activity in the delta (slow wave) band (0.5 Hz to 4.0 Hz), typically averaged separately for NREM and REM sleep for the entire night.
**Slow wave sleep:**	The deepest stage of NREM sleep (stage N3), characterized by more than 20% delta wave EEG activity.
**Stage N1:**	The lightest stage of sleep, which occurs right after falling asleep; characterized by low-voltage, fast EEG activity.
**Stage N2:**	The intermediate stage of sleep that follows stage N1; characterized by theta activity (4–7 Hz), K-complexes, and bursts of faster activity on EEG.
**Stage N3:**	The deepest stage of sleep; characterized by high-amplitude slow waves on EEG.
**Total sleep time:**	The total number of minutes asleep.
**Total wake time:**	The total number of minutes awake during the sleep period.
**Wake after sleep onset:**	The number of minutes awake after falling asleep.

Historically, studies of AUD and sleep have mostly included men. Although women with AUD have been recruited for a handful of studies,^3^–^7^ women have largely been underrepresented in the research that examines the relationship between sleep and alcohol use. Sex differences in the effects of alcohol are dependent on the interaction of many biopsychosocial factors. Sleep intertwines with several of these relationships: alcohol disrupts sleep, and sleep disturbance relates to increased risk of psychiatric co-occurrence, alcohol misuse, and relapse to AUD. In addition, sleep is a modifiable behavior.^8^,^9^ Thus, understanding how sleep problems relate to problematic alcohol use and the extent to which this relationship differs between men and women can inform the development of targeted methods for prevention and treatment of AUD.

This narrative review aims to stimulate new research in this area by consolidating findings from studies that have reported effects of acute and chronic use of alcohol on sleep among women. First, an overview of sex differences in sleep disorders is provided, followed by considerations for how sex may modify the relationship between alcohol use and sleep. (For consistency, both biological and psychological/sociological/cultural factors are referred to as “sex”-related throughout the review.) The review concludes by providing treatment considerations and directions for future research.

## SEX DIFFERENCES IN SLEEP

Sleep is a universal process across species and is a behavioral state that is essential to physical and mental health in humans. Changes in brain activity throughout the night demarcate different stages of sleep. This neuronal activity, along with muscle activity and eye movements, can be measured via polysomnography (PSG) to provide an objective measure of sleep. Sleep is divided into stages (N1, N2, and N3) of non–rapid eye movement (NREM) sleep and rapid eye movement (REM) sleep.^10^ Throughout the night, sleep follows a cyclical pattern. Each cycle begins with stage N1, and the majority of time is spent in stage N2 before progression to stage N3 (deep sleep) and eventually to REM sleep. Each cycle lasts approximately 90 minutes. More detailed analysis of the sleep electroencephalogram (EEG) is possible with spectral analysis to determine activity during sleep within a specific frequency band (e.g., slow wave activity).

PSG provides a detailed, objective measure of sleep architecture and quality but is mainly confined to the laboratory. Actigraphy (usually measured with devices worn on the wrist) relies on an accelerometer to measure patterns of activity from which sleep–wake states can be estimated.^11^ Actigraphy is useful for objective assessments of sleep outside the laboratory environment. Self-perception of sleep quality is also valuable and can be measured over many nights with questionnaires or sleep diaries.

### Differences in Sleep Measures

Women tend to have better sleep quality, as measured by PSG, than men. Women have less total wake time, shorter sleep onset latency, better sleep efficiency, and a larger percentage of slow wave sleep and slow wave activity **(**for definitions of these sleep measurements, see the box** Glossary of Sleep Terms**).^12^ The prevalence of sleep-disordered breathing is 9% among women versus 24% in men. However, women with sleep-disordered breathing are more likely to present with initial symptoms of insomnia or fatigue rather than the typical symptoms associated with sleep-disordered breathing, such as snoring, daytime sleepiness, and witnessed apneic events.^13^

Although PSG is considered the gold standard of sleep measurement, it has limitations. PSG cannot capture habitual sleep duration under naturalistic settings and may miss subcortical brain activity (particularly in regions shown to be involved in conscious awareness) that may be more prominent in individuals with insomnia than in those who sleep well.^14^ Although not yet examined, possible sex differences in subcortical brain activity during sleep may explain the finding that women report poorer subjective sleep quality than men despite having better PSG-based sleep quality.

When using subjective measures, women report more sleep problems than men, including disrupted and insufficient sleep, poor sleep quality, difficulty falling asleep, frequent night awakenings, and time awake during the night.^15^,^16^ Women also have a 40% greater risk of insomnia^12^ and report earlier sleep timing (i.e., bedtime and wake time) than men.^17^ Potential reasons for sex differences in sleep are described briefly in this review. For more detailed discussions, see the reviews by Mong and Cusmano^12^ and Krishnan and Collop.^13^

### Biological Differences

Sex steroids (i.e., testosterone in men and estrogen and progestins in women) modulate sleep differently. Generally, women’s sleep is more sensitive to changes in ovarian steroids.^12^ For example, sex hormones modulate the orexin/hypocretin system, which plays an important part in regulating sleep and wake states.^18^ Therefore, fluctuations in ovarian steroids in women (e.g., puberty, menstrual cycle, menopausal transition) are associated with changes in sleep and circadian rhythms^19^ and increased prevalence of sleep disturbance.^20^,^21^ In addition, among men and women with similar sleep timing and duration, women have a shorter circadian period and earlier circadian timing of endogenous temperature and melatonin rhythms.^12^ (For definitions of these circadian terms, see the box** Glossary of Sleep Terms.**) This mismatch in sleep timing and circadian timing can cause sleep disturbance, such as problems with sleep maintenance and/or early morning awakening, which, in part, may underlie women’s increased risk for insomnia.

### Psychosocial Differences

Among women, those with more anxiety and more perceived nighttime awakenings also report worse subjective sleep quality, despite a lack of objectively measured sleep disturbance.^12^ Anxiety and depression are both more prevalent among women and are strongly associated with insomnia. The risk of affective disorders increases at the onset of puberty, especially among girls.^22^

## ALCOHOL AND SLEEP

Sex differences occur in sleep continuity and sleep architecture measures as well as in the prevalence of sleep disorders like insomnia and obstructive sleep apnea. Sex differences also have been reported in alcohol use patterns, biological effects of alcohol, and risk factors for heavy alcohol use. Alcohol use likely affects sleep systems differently in men and women, and pathways that link sleep disturbances with subsequent heavy alcohol use also may differ according to sex. In this section, we review the evidence for sex differences in bidirectional relationships between sleep quality and alcohol use (although directionality is not always clear when based on findings from observational or cross-sectional studies).

Sleep and wake states are regulated by complex patterns of neurotransmitter release and neural activation, many of which are affected by alcohol.^23^ Individuals who have trouble sleeping may initiate alcohol use as a sleep aid. Because alcohol affects the gamma-aminobutyric acid (GABA) neurotransmitter system, alcohol acts as a sedative and reduces time to sleep onset, increases slow wave sleep, and suppresses REM sleep in the first half of the night.

Alcohol has acute neurotoxic effects that affect receptors important for sleep generation. As alcohol metabolizes (at 7 grams per hour, on average), its sedating benefits diminish.^24^ Later in the night, sleep becomes more disrupted and awakenings are more frequent. Thus, the effects of alcohol on sleep differ depending on which half of the night is examined. Chronic alcohol exposure damages nerve cells and fibers, reducing the likelihood of synchronized neuronal firing across the cortex, which is necessary for slow wave sleep. With prolonged use, neurotransmitter systems adapt and modulate their release, which can increase sleep disruption and change sleep architecture, sometimes permanently.^23^,^25^

Studies (mostly among men) indicate that these changes in sleep structure persist during abstinence, and disturbed sleep is a risk factor for relapse.^1^ Therefore, sleep disturbance has been suggested as a target for treatment, potentially decreasing the risk of problematic alcohol use while also increasing the likelihood of abstinence.

### Sleep Architecture

This section examines studies (which included women participants) of both the acute and chronic effects of alcohol on sleep architecture. To the extent possible, results from experimental studies are emphasized.

### Effects of acute alcohol use

First, we present studies that primarily used PSG to examine the acute effects of alcohol on sleep architecture. These experiments provide some evidence of directionality in the relationship between alcohol use and subsequent sleep quality.One of the first studies to investigate the effect of acute alcohol use on sleep, specifically in young women, was conducted by Williams and colleagues.^26^ As part of this double-blind trial, 11 healthy women (ages 18 to 21) completed several nights of PSG an hour after consuming a beverage with either 0.00, 0.50, or 0.75 grams of alcohol per kilogram of body weight (g/kg). Results were consistent with previous findings reported for men. As the alcohol dose increased, sleep onset latency decreased. A significant decrease in the percentage of REM sleep was found, which was most apparent in the first 3 hours of the night. Also, a dose-dependent increase in slow wave sleep during the first half of the night was found, followed by a decrease in slow wave sleep in the second half of the night. Furthermore, these women demonstrated a dose-dependent increase in the percentage of stage N1 sleep, with increased minutes spent in stage N1 sleep in the second half of the night.

A later study conducted by Van Reen and colleagues examined the extent that a moderate dose of alcohol (0.49 g/kg), compared to placebo, consumed an hour before bedtime affected the sleep and sleep EEG of 7 women (ages 22 to 25).^27^ Similar to the findings reported for men,^23^ this study reported that alcohol consumption led to an increase in slow wave sleep (in the first 2 hours) and an overall decrease in REM sleep.^27^ Also, frontal EEG power during NREM sleep in the alpha range (9 to 11 Hz) increased relative to placebo following alcohol consumption.

In a direct evaluation of sex differences, Arnedt and colleagues performed PSG for 93 healthy adults (ages 21 to 31, 59 were female) following alcohol intoxication.^28^ For this double-blind, randomized trial, all participants received alcohol on one night and placebo on another night, 1 week apart. Participants were given either placebo or alcohol (1.2 g⁄kg for men and 1.1 g⁄kg for women) 1 to 2.5 hours before bed. The alcohol dose was adjusted for weight and sex such that breath alcohol concentration (BrAC) levels were equivalent in men and women. At bedtime on the alcohol night, women reported higher ratings of sleepiness than men. Despite reaching equivalent BrACs, sleep continuity was more disrupted in women than in men. For women, the total sleep time decreased by 20 minutes relative to the placebo night, and the wake after sleep onset time increased by 15 minutes. In addition, among women participants, the frequency of awakenings increased, and overall sleep efficiency decreased by 4% after alcohol intoxication. In men, no significant differences in sleep continuity measures (i.e., sleep onset latency, total sleep time, sleep efficiency, frequency of nighttime awakenings, and wake after sleep onset) between the placebo and alcohol conditions were reported. For both sexes, sleep architecture variables differed for the alcohol condition compared to the placebo condition—alcohol use increased slow wave sleep and decreased REM sleep.

Chan and colleagues also examined the effects of acute alcohol consumption (a mean dose of 0.828 g/kg an hour before bedtime) on the sleep architecture of 24 older adolescents (ages 18 to 21, 12 were female).^29^ They found main effects of alcohol on sleep, dependent on halves of the night. In the first half of the night, participants experienced fewer arousals, less stage N1 sleep, increased slow wave sleep, and reduced REM sleep. In the second half of the night, they experienced less sleep efficiency and more time awake after sleep onset. These researchers did not find evidence for an interaction between sex and alcohol.

### Effects of chronic alcohol use

The following studies are observational, such that they examine sleep among individuals with a history of chronic alcohol use in the context of many other variables. Individuals in these studies vary regarding the duration of their abstinence at the time of study, their co-occurring disorders, and their lifetime alcohol use. When participants were examined early (at less than 1 month) during recovery, the effects on sleep may have reflected the effects of withdrawal more than any chronic effects of heavy alcohol use. When participants were examined later during recovery, withdrawal effects would have subsided. Therefore, the associations observed do not prove causality in these relationships, but they provide a starting point to stimulate further research that may better distinguish directionality.

Colrain and colleagues collected sleep architecture and EEG measures from 42 abstinent participants (mean age of 49, 15 were women) with long-term AUD and from 42 control participants (mean age of 51, 23 were women).^5^ Overall, women had better sleep efficiency, fewer periods of in-bed awake time, and more slow wave activity during NREM sleep than men. There were main effects of AUD for some sleep measures. For example, individuals with AUD had less slow wave sleep and slow wave activity during NREM sleep and more stage N1 and REM sleep than controls.

Despite a lack of significant interaction between sex and diagnosis, women with AUD and women control participants had similar amounts of NREM slow wave activity, whereas men with AUD had substantially lower NREM slow wave activity than men control participants.^5^ Women with AUD had lower levels of lifetime alcohol consumption and longer periods of sobriety when compared with the men who had AUD in this study. Although greater estimated lifetime alcohol consumption was related to a lower percentage of slow wave sleep in men, this measure was not related to the percentage of slow wave sleep in women. This study did not investigate sex interaction effects, and the samples of women and men with AUD were unequal sizes, had varying lengths of sobriety, and had different levels of lifetime alcohol exposure.

Using the same sample, Colrain and colleagues examined K-complex incidence and amplitude during sleep.^6^ K-complexes are high-voltage, delta frequency events that occur during NREM sleep when large numbers of healthy neurons fire together at the same time. They provide a sensitive measure of typical, healthy, brain aging. In this study, participants with AUD had both reduced K-complex incidence and amplitude. Men and women also showed the same pattern of AUD-related change in K-complex amplitude, despite women having less lifetime alcohol consumption.

In a sample that included 26 participants (ages 32 to 63, 10 were women) with alcohol dependence who were in subacute withdrawal from alcohol and 23 control participants (ages 24 to 61, 9 were women), overall, women spent a larger proportion of time awake during the sleep period, and they had shorter time to REM sleep.^7^ The relationships between sleep parameters and group did not vary by sex; however, this analysis may have been underpowered because of the sample size. The investigators noted that the distribution of sex across groups was not equal.

A population-based study of sleep among 400 Swedish women (ages 20 to 70) found that women who self-reported alcohol dependence had longer sleep onset latency, reduced REM sleep, and more stage N2 sleep compared to women who did not report alcohol dependence.^30^ In addition, alcohol dependence was related to decreased time spent in REM sleep and increased sleep onset latency, independent of age, body mass index, apnea-hypopnea index, smoking, and hypertension.

#### Summary

Sleep is a complex neurological function, and the extent that it may be affected after a single night of alcohol compared to chronic alcohol misuse can differ. Thus, sex differences in the acute effects of alcohol may not necessarily coincide with sex differences in the chronic effects of alcohol. The single experimental study that examined sex differences in the effect of acute alcohol consumption found sex differences in objectively measured sleep among healthy subjects (with equivalent BrAC levels before sleep), with women showing more disrupted sleep than men.^28^

Sex differences in alcohol pharmacokinetics may underlie these differences. Even at equivalent starting points, BrAC levels decline more rapidly for women than for men.^28^ As alcohol metabolizes, alcohol metabolites disrupt sleep. Chronic alcohol misuse leads to changes in brain macrostructure and microstructure that can manifest as sleep disturbance.^25^ Few studies have examined sleep in both men and women during recovery from AUD, and those studies have not had sample sizes large enough to statistically examine sex differences.

Further study is needed to examine potential sex differences in sleep among individuals with AUD who are abstinent. Dose effects, time in recovery, and the effects of interaction between age and sex should be considered. Sleep structure changes across age, and these changes vary by sex.^31^ For example, women have a greater amount of slow wave activity than men, and although men tend to show a decrease in slow wave activity with age, women do not show the same pattern of decline.^12^

### Sleep Physiology

Limited experimental work has examined whether the effects of alcohol on the functioning of physiological systems (e.g., respiratory or cardiovascular) during sleep differ according to sex.

#### Effects of acute alcohol use

In an investigation of the acute effects of alcohol, Block and colleagues monitored breathing and oxygenation during sleep for 78 participants (20 were men ages 20 to 40 years, 20 were men ages 40 years and older, 20 were women ages 20 to 40, and 18 were postmenopausal women ages 51 to 66) following consumption of 2 milliliters of alcohol per kilogram of body weight.^32^ Men in both groups had more oxygen desaturation episodes across the night and greater severity of desaturation, but no effect of alcohol on breathing or oxygenation was found for either group of women. As expected, postmenopausal women had significantly more episodes of apnea and oxygen desaturation than premenopausal women, although this difference was unrelated to alcohol consumption.

A large, observational study of 1,420 men and women (mean age of 51, 645 were women) demonstrated similar findings.^33^ Men showed increased likelihood of sleep-disordered breathing for each drink consumed per day (measured via a self-report questionnaire), whereas no association between minimal to moderate alcohol consumption and sleep-disordered breathing was found for women. The investigators posited that circulating progesterone may protect young women in particular from the depressant effects of alcohol and consequent sleep apnea and oxygen desaturation,^32^,^34^ and that hormonally mediated increased ventilatory drive and anatomical differences may also protect women from sleep-disordered breathing events.^33^,^35^,^36^ Since alcohol had no effect on breathing for postmenopausal women, other nonhormonal factors may have played a role in the sex differences related to sleep-disordered breathing and alcohol consumption.

#### Effects of chronic alcohol use

A study of 24 patients with chronic AUD who were recently abstinent (10 were women ages 25 to 58) compared with 24 control participants (10 were women ages 25 to 58) showed that both males and females with AUD had a high number of observed apneic/hypopneic episodes, and this result did not differ by sex.^37^ The researchers concluded that women with AUD were as likely as men with AUD to have a sleep-related breathing disorder.

In a study investigating autonomic nervous system functioning during sleep, de Zambotti and colleagues found that patients with AUD who were recently sober (*n* = 14, 7 were women ages 28 to 54) compared with healthy control participants (*n* = 16, 8 were women ages 30 to 62) had elevated heart rates, reduced total heart rate variability, and reduced high-frequency activity (a measure of vagal functioning) across the night.^4^ Together, this pattern of findings indicates disrupted autonomic nervous system functioning during the night, providing compelling evidence of impaired cardiovascular functioning during sleep. Effects did not differ by sex, and women with AUD, despite having less lifetime alcohol consumption, were affected to the same extent as men with AUD. In a follow-up investigation across the first few months of abstinence, as the duration of abstinence increased, individuals with AUD showed substantial recovery in heart rate and vagal functioning during sleep, although examination of any modifying effect by sex was not possible in this small sample.^3^

Periodic limb movements can also contribute to disturbed sleep. Aldrich and Shipley found that periodic limb movements were more likely to occur at a clinically significant frequency among adults ages 19 to 81 who self-reported consuming 2 or more drinks per day (heavy users, *n* = 112, 24 were women) when compared with adults who consumed less than 2 drinks per day (abstainers and light to moderate users, *n* = 872, 317 were women).^38^ In addition, women who were heavy users were more likely to report symptoms of periodic limb movements than women who were light users, whereas no difference was observed between the two groups of men.

#### Summary

For physiological measures, the evidence from one large, experimental study suggests that acute alcohol consumption does not affect women’s breathing during sleep to the same extent it does for men, who demonstrate more oxygen desaturation events during the night. Also, among men, self-reported alcohol use is positively associated with greater likelihood of sleep-disordered breathing, although this relationship is not observed in women. However, women with AUD are just as likely as men to have sleep-disordered breathing.^37^

Women may be more susceptible to periodic limb movements, and alcohol use could be a potential trigger of these movements. Also, women who experience periodic limb movements may self-medicate with alcohol. One study with a small sample size suggested that chronic alcohol use may affect cardiovascular functioning in women more than it does in men, as women and men did not differ in these measures despite women having less lifetime alcohol consumption.

These results are consistent with other studies that have demonstrated that women are at greater risk of alcohol-induced cardiomyopathy and peripheral neuropathy despite fewer years of drinking and lower quantities of alcohol consumption.^39^ Given that two of these studies examined men and women early during their recovery,^4^,^37^ some of the effects found could reflect residual withdrawal effects of alcohol. Further longitudinal studies across a period of recovery among men and women with AUD are needed to separate effects of alcohol withdrawal and chronic heavy alcohol use on sleep as well as on physiological measurements taken during sleep.

### Self-Reported Sleep Behavior

Many individuals report using alcohol as a sleep aid,^40^,^41^ even though the use of alcohol to help initiate sleep can further perpetuate sleep disturbance. In women older than age 60, using alcohol to sleep and shorter sleep onset latency each are associated with greater risk for alcohol misuse.^42^ However, moderate alcohol use is associated with fewer insomnia symptoms in women, but not in men, older than age 65.^43^

In a study of healthy men and women, self-reported insomnia symptoms at baseline were associated with greater odds of heavy drinking at a 5-year follow-up.^44^ Likewise, heavy drinking and binge drinking at baseline were associated with greater odds of insomnia symptoms at a 5-year follow-up. Although results specific to sex were not reported, the investigators noted that these associations were similar among men and women but reached statistical significance only for women.

Some epidemiological studies have considered associations between alcohol use and insomnia symptoms among women in midlife and after menopause, an age group in which sleep problems are common. Blümel and colleagues reported that troublesome drinking (assessed with the Brief Scale of Abnormal Drinking) in a group of women ages 40 to 59 was strongly associated with increased risk for insomnia symptoms more than other factors, including mood and vasomotor symptoms, education level, and use of hypnotics.^45^ In contrast, frequency of alcohol use (i.e., not currently, occasionally, or regularly in the past week) was not associated with sleep disturbances in a group of postmenopausal women (*N* = 322, ages 60 to 70).^46^ These findings show that relationships between alcohol use and insomnia for women may differ depending on whether frequency of alcohol use or troublesome drinking are examined.

A large, longitudinal study of 9,941 Norwegian adults (53.6% were women) found that men reporting high levels of alcohol consumption at baseline were at higher risk of reporting sleeplessness at a follow-up 13 years later.^47^ Similarly, men who experienced sleeplessness at baseline also were at higher risk of reporting high levels of alcohol consumption at the follow-up, demonstrating the bidirectionality of associations between sleep problems and alcohol use. In contrast, no such relationships were found for women.

A population-based study of 3,450 French adults (52.4% were women ages 18 to 64) reported that drug use for insomnia (prescription or nonprescription) was associated with alcohol misuse among men but not among women.^48^ The only study of insomnia prevalence among individuals in treatment for AUD found that women and men reported similar rates of insomnia symptoms, despite a larger prevalence of insomnia among women in the general population.^49^ Also, insomnia symptoms at baseline were significantly associated with relapse to AUD for both men and women.

The extant data are mixed regarding whether women show differential risk for associations between self-reported sleep disturbance and alcohol use. However, these observational studies, which rely entirely on self-report methods to measure both alcohol use and sleep disturbance, use different questionnaires and, in some cases, use measures limited to a single item. More research is needed to characterize the relationship between sleep behavior and alcohol use among women, especially studies that help distinguish sleep problems as predictors of relapse and alcohol use as a predictor of insomnia. Further investigation should use more comprehensive, frequent measures of sleep behavior (e.g., sleep diaries) potentially combined with objective measures (e.g., actigraphy) and measures of alcohol consumption to better characterize sex differences in these relationships.

### Sleep as a Predictor of Adolescent Alcohol Use

As early as childhood, self-reported sleep problems are related to onset of substance use in adolescence.^50^ In the first prospective study of sex differences in this relationship, Wong and colleagues found that sleep problems in childhood were a significant predictor of onset of drinking in both boys and girls but at earlier ages for boys (8 to 14) than girls (15 to 17).^51^ In a large, community-based sample of 7,507 children and adolescents in Hong Kong (48.5% were females ages 6 to 17), Zhang and colleagues found that boys with insomnia symptoms were more likely to report regular consumption of alcohol (sometimes or often), whereas no such relationship was found for girls.^52^

In a population-based study of 4,187 Finnish adolescents (51.8% were females ages 11 to 15), perceived tiredness was related to increased likelihood of drinking and smoking for boys, but for girls it was only related to an increased likelihood of smoking.^53^ In contrast, in a large sample of 13,381 U.S. adolescents (48.8% were females ages 12 to 17), there was a stronger relationship between subjective sleep problems and substance use in general (i.e., use of cigarettes, alcohol, or illicit drugs) for girls than for boys.^54^

Unpublished data from Hasler and colleagues (2017) suggest that in a sample of 729 adolescents (368 were females ages 12 to 21) from the National Consortium on Alcohol and Neurodevelopment in Adolescence (NCANDA) study, females with worse sleep quality were more likely to report binge alcohol use at baseline. However, males with worse sleep quality at baseline were at a greater risk of worsening binge alcohol use a year later.

Emerging data from longitudinal studies that track sleep patterns in adolescents before the onset of alcohol use suggest there may be sex differences in the relationships between sleep behaviors and alcohol use.^50^ However, further data are required before definitive conclusions can be reached. Such work is needed to determine sex differences in the directionality of the relationships between substance use and sleep and circadian factors, as well as the underlying mechanisms of these relationships.

### Sleep and Circadian Timing

Circadian rhythm disturbance can underlie sleep problems, and alcohol use alters many circadian functions (e.g., blood pressure, body temperature, hormone release).^55^ Proper assessments of melatonin level, cortisol level, or body temperature, which are validated methods for measuring circadian rhythm, require rigorous laboratory protocols conducted over multiple hours to days and, thus, are not always feasible. Measurements of circadian preference (i.e., morningness-eveningness), chronotype, or sleep timing can serve as proxies for direct measures of circadian patterns of sleep–wake activity. To our knowledge, no studies have directly examined whether sex moderates the relationship between alcohol use and circadian rhythms in humans. One preclinical study that used mice with a knockout of adenosine equilibrative nucleotide transporter type 1 (ENT1), which is associated with both AUD and circadian/sleep disruptions, showed that circadian rhythm disruption increased alcohol consumption in male but not female mice,^56^ suggesting that further investigation of sex differences in this area is warranted in humans.

Although more bona fide circadian research is needed, proxies for circadian rhythm, such as eveningness and late chronotype, consistently are associated with more alcohol use and problems with alcohol.^57^ On average, women tend towards a relatively earlier sleep and activity pattern (i.e., morningness/early chronotype), which theoretically might lower the risk of alcohol use associated with circadian factors.

Hasler and colleagues investigated the effect of sleep timing on response to alcohol among 148 young adults (50 were women ages 21 to 35).^58^ In males (White males only) but not in females, later sleep timing and greater eveningness preference were associated with a greater self-reported stimulating effect of alcohol immediately following alcohol consumption. In addition, greater variability in sleep duration was related to greater sedation following alcohol consumption for both men and women. Further work is needed to examine links between circadian factors and heavy alcohol use, particularly among adolescents, to establish potential sex-specific predictors of alcohol use.

## CLINICAL CONSIDERATIONS AND TREATMENT

Some sleep abnormalities may predate the effects of alcohol and also may differ between men and women. In addition, the prevalence of different sleep disorders must be taken into consideration. As already described, women are 40% more likely to develop insomnia than men.^20^ Individuals may be vulnerable to the development of insomnia for a variety of reasons.^1^ Predisposing factors such as genetics (e.g., *CLOCK* gene polymorphism or family history of AUD), childhood trauma, and childhood sleep problems increase an individual’s risk of developing insomnia. Precipitating factors are stress-promoting events that trigger acute insomnia. Perpetuating factors are maladaptive compensatory behaviors, such as reading in bed or drinking alcohol, used to cope with sleep difficulty. Screening women for sleep problems may help providers intervene before problematic use of alcohol develops or may increase the likelihood of maintaining abstinence.

Pathways toward alcohol use vary developmentally, and sleep characteristics during childhood and adolescence predict risk for onset of alcohol use and misuse.^59^ Childhood sleep problems are related to the onset of alcohol use in adolescence; therefore, treating sleep problems early in life may confer some benefit by delaying the onset of alcohol use. Furthermore, sleep disorders often manifest during reproductive transitions (e.g., puberty, pregnancy, menopause).

Females tend to develop insomnia after puberty, and the later sleep timing that occurs during puberty is positively associated with alcohol use.^16^ Addressing the sleep disturbances of pregnant women is especially important. Alcohol consumption during pregnancy acutely affects fetal sleep behavior, and research suggests that prenatal alcohol exposure is related to persistent sleep disruption in affected children.^60^ For many women, sleep disturbance and complaints of insomnia increase during and after the menopause transition.^12^ The sleep changes related to aging, hormonal fluctuations, and psychological adjustment may contribute to women in this age group being particularly vulnerable to developing AUD.^61^

Improved understanding of the mechanisms by which these hormones modulate sleep may help guide development of novel therapies for treatment of problematic alcohol use. Such studies will help health care providers make informed decisions about medications (and dosages) and behavioral interventions that will be effective for treating sleep problems among women with AUD.

Cognitive behavioral therapy for insomnia is the first line of treatment for insomnia and is equally effective for men and women.^8^,^62^ This nonpharmacological treatment method focuses on behaviors, cognitions, and associations that contribute to poor sleep.^63^ The therapy uses a combination of sleep restriction (i.e., limiting time spent awake in bed), stimulus control, sleep hygiene (that is, healthy sleep habits such as consistent bed and wake times, comfortable bedroom environment, or avoiding caffeine and alcohol before bedtime), and cognitive therapy to address distorted beliefs about sleep. Up to 80% of patients benefit from this therapy, and treatment effects are maintained at follow-up a year later.^9^ Pharmacotherapy is the next evidence-based approach for treatment of sleep disturbance, and it often is used in conjunction with cognitive behavioral therapy for insomnia, although it can be contraindicated for individuals with AUD.

Although women tend to have better long-term treatment outcomes than men, they are less likely to receive services specifically for alcohol-related issues, and they are more likely to seek treatment in settings that are not alcohol specific.^39^ Educating health care providers in the primary care setting to screen women for AUD and sleep problems may help reduce the stigma many women face when seeking appropriate treatment for AUD.

In addition, management of sleep problems is not typically a first line of treatment for individuals with AUD, despite the association between insomnia symptoms and increased risk of relapse. Sleep is a modifiable behavior that, if improved, may have downstream benefits for other health outcomes.^23^ Medication trials (e.g., trazodone, gabapentin, quetiapine) have shown mixed efficacy and can be contraindicated in individuals with AUD, whereas behavioral treatments for insomnia consistently have been more effective in treating sleep problems, with moderate to large effect sizes.^1^

Treating sleep problems early may reduce risk for subsequent AUD. Considering that for women depressive symptoms predict alcohol consumption, cognitive behavioral therapy for both insomnia and depression may help prevent problematic alcohol use with two points of intervention. Although cognitive behavioral therapy for insomnia has not been shown to differentially improve alcohol outcomes,^64^,^65^ more randomized controlled trials are warranted. This therapy has already shown promise as a treatment for insomnia among individuals with AUD, and men and women with no AUD respond to the therapy equally well.^66^ It will be valuable for future studies to investigate the utility of cognitive behavioral therapy for insomnia and of other treatments that aim to improve sleep in individuals with AUD, as well as to examine whether these treatments are equally effective in men and women.

## FUTURE DIRECTIONS AND CONCLUSION

Suggested areas for future research on sex differences related to alcohol and sleep include examination of:

- Alcohol’s neurotoxic effects on circuits important for sleep generation
- Sleep during sustained abstinence from alcohol
- Cardiovascular functioning at night following alcohol use
- Alcohol use and its relationships with circadian misalignment and shiftwork
- Hormonal change and reproductive phase (e.g., puberty, the menstrual cycle, pregnancy, menopause) effects on alcohol use and sleep
- Other demographic factors (e.g., age, race, ethnicity, socioeconomic status) and how they affect alcohol use and sleep
- Longitudinal studies of sleep before initiation of alcohol use and across the course of recovery in individuals with AUD who are abstinent
- Cognitive behavioral therapy for insomnia and other treatment efficacy and effectiveness in improving sleep for individuals with AUD

Women historically have been underrepresented in research studies on alcohol use and sleep. Although AUD currently is more prevalent among men, the male/female differences in patterns of alcohol consumption are converging. Now, more than ever, sex differences need to be considered in all aspects of alcohol research. Only a small body of literature has investigated sex differences or interactions with sex in relation to sleep outcomes and alcohol use, making it challenging to draw definitive conclusions from the research thus far. Sleep and alcohol use vary by race and ethnicity,^67^ and further research examining these characteristics in the context of sex differences is needed.

In addition to understanding sex differences in the relationship between alcohol and sleep, understanding the consistencies in the effects of alcohol on sleep among men and women is important. Alcohol has the same detrimental effects on many aspects of sleep and sleep physiology, regardless of sex. Given that sleep disturbance is so commonly reported by individuals with AUD, and the strong associations among sleep, daytime functioning, and mental and physical health, understanding how these relationships might differ in women compared to men is crucial to developing targeted and appropriate treatment recommendations.
